# Community pharmacists’ views and experiences toward over-the-counter medicines misuse and abuse in Saudi Arabia: A qualitative study

**DOI:** 10.3389/fphar.2022.997342

**Published:** 2022-11-28

**Authors:** Mohammad Algarni, Zahraa Jalal, Muhammad Abdul Hadi, Saleh Alghamdi

**Affiliations:** ^1^ School of Pharmacy, Institute of Clinical Sciences, College of Medical and Dental Sciences (CMDS), University of Birmingham, Birmingham, United Kingdom; ^2^ College of Pharmacy, QU Health, Qatar University, Doha, Qatar; ^3^ Department of Clinical Pharmacy, Faculty of Clinical Pharmacy, Al Baha University, Al Baha, Saudi Arabia

**Keywords:** community pharmacist, over-the-counter, medicines, misuse, abuse

## Abstract

**Background:** Community pharmacists are uniquely positioned to identify and address the issue of misuse and abuse of over-the-counter (OTC) medicines. To date, no study has explored the Saudi community pharmacists’ views and experiences regarding aspects of OTC medicines’ misuse and abuse.

**Objective:** To explore the views and experiences of the Saudi community pharmacists towards OTC medicines misuse and abuse. Furthermore, we aimed to identify frequently misused and abused medicines, the reasons and contributing factors, the role of pharmacists, and potential risk-mitigating strategies.

**Methods:** Semi-structured interviews were conducted with a convenient sample of sixteen community pharmacists recruited from community pharmacies across the AL-Baha region, Saudi Arabia. Interviews were conducted using a pilot-tested interview guide in the Arabic language. All interviews were audio-recorded, transcribed verbatim, translated from Arabic into English, and then thematically analysed.

**Results:** Analysis of interviews generated five main themes, including the commonly misused and abused OTC medicines, reasons and factors contributing to misuse and abuse of OTC medicines, pharmacists’ interventions to manage misuse and abuse, challenges and barriers to pharmacists’ interventions in misuse and abuse issues; and potential strategies to reduce the risk of OTC medicines misuse and abuse and improve pharmacists’ practice. Sedative antihistamines, cough products containing dextromethorphan, codeine-based analgesics, and non-codeine-based analgesics were commonly misused and abused OTC medicines. Managing ongoing medical conditions was the main reason for misusing OTC analgesics while recreational use and inducing sleep were the common reasons for abuse. Several factors contributing to misuse and abuse were reported, including unprofessional advice sought from other people, lack of awareness about medicines, and commercial advertisement of OTC products. Community pharmacists identified misuse and abuse among customers by judging their behaviours and attitudes and using structured questioning techniques. Counselling customers on the appropriate use of medicines, providing safe alternatives, and refusing to sell products were among the commonly used actions of pharmacists to address misuse/abuse. Pharmacists proposed several strategies to reduce the risk of OTC medicines misuse/abuse but believed that rescheduling OTC medicines with abuse potential to prescription-only medicine was the best option.

**Conclusion:** Community pharmacists believed that the misuse and abuse of OTC medicines amongst pharmacy customers was common. A multidimensional strategy consisting of upskilling community pharmacists, a comprehensive review of OTC medicines sale regulations, and patient education to limit the risks of OTC medicines misuse/abuse is required.

## Introduction

Over-the-counter (OTC) medicines, also known as non-prescription medicines, can be obtained without a prescription from a physician. They are frequently used in the prevention and treatment of various conditions such as common cold, headache, musculoskeletal pain, and heartburn (Bowman et al., 2020). Although they provide benefits to patients, their use is not without risks. These risks may include improper patient understanding of the underlying condition, wrong dosage, dependence or addiction, adverse reactions, and drug-drug interactions (Wazaify et al., 2005). Unlike prescription medicines, OTC medicines are safe in general when used as recommended, but there is potential for misuse and abuse as well. Misuse of medicines refers to the use of OTC medicines for medical purposes but inappropriately, such as taking higher doses than recommended (Cooper, 2013b). However, abuse refers to the use of medicines for non-medical reasons such as recreational purposes (Hall et al., 2012). As community pharmacists are often the primary contact for patients requiring OTC medicines, they can take proactive actions in preventing and managing misuse and abuse issues by exploiting their clinical skills, offering oral and written medicine information, and establishing trust among patients (Roussin et al., 2013). However, pharmacists face multiple challenges in identifying and managing the problematic use of OTC medicines such as a lack of access to patient medication history, making patient counselling challenging (Griese et al.).

Unlike other countries, OTC medicines are not available in Saudi Arabia for sale in supermarkets or groceries, and their provision is restricted to only community pharmacies. A number of studies have been undertaken in Saudi Arabia exploring the public’s attitude toward OTC medicines. Al-Khamees et al. (2018) reported that OTC analgesics were the most commonly used OTC medicines with diclofenac being the most commonly used analgesic. A study estimating the prevalence of OTC medicine misuse among Saudi female university students reported the lifetime prevalence of misuse to be 29.1%. Diphenhydramine and Paracetamol were the most frequently misused medicines while pain relief and inducing sleep were the main reported reasons (Dabbagh et al., 2021). In another study, 6.6% of the study participants reported daily use of OTC analgesics (Qahl et al., 2020).

Since community pharmacists are the only point of contact for individuals seeking OTC medicines, they can play an important role in detecting and managing misuse/abuse incidents. To date, no study has sought to explore community pharmacists’ views and experiences on various aspects of OTC medicine’s misuse and abuse. Therefore, this study aimed to explore the views and experiences of community pharmacists in Saudi Arabia towards OTC medicines misuse and abuse, particularly to identify: OTC medicines commonly misused/abused, reasons and contributing factors, pharmacists’ roles in identification and management, barriers to pharmacists’ interventions and potential strategies to reduce the risks of OTC medicines misuse/abuse and improve pharmacists’ current practice to ensure safe and effective use of OTC medicines.

## Methods

### Design

This was an exploratory qualitative study using in-depth semi-structured interviews. The use of semi-structured interviews as a data collection method allowed researchers to gain spontaneous expressions from participants, minimise pre-conceptualised boundaries, and stay close to the study objectives (Smith, 1998).

### Setting

The research was conducted among community pharmacists working at a national pharmacy chain (X) across different locations in the Al Baha region, Saudi Arabia. The participating chain pharmacy is one of the largest national chains in Saudi Arabia, operating more than 1,000 pharmacies. Al Baha is one of the thirteen regions of Saudi Arabia and is located in the Southwest of the country. There are 96 community pharmacies across this region (Saudi Ministry of Health, 2022). Pharmacists working in other pharmacy chains and independent pharmacies in the region were also approached, but none responded to our invitation to participate in the study.

### Interview guide development

The Interview guide was developed based on the study objectives, the reviewed literature, and discussion among the research team (Cooper, 2013a; Algarni et al., 2021). The interview guide comprised open-ended questions and further probing questions to seek information about participants’ characteristics and perspectives towards various aspects of OTC medicines misuse/abuse. These aspects involved the commonly implicated medicines in misuse/abuse and pharmacists’ experiences with recent misuse/abuse cases, reasons, and factors contributing to misuse/abuse, methods of identifying and addressing misuse/abuse incidents, barriers to pharmacists’ interventions, and potential strategies to minimise the risk of OTC medicines misuse/abuse and improving pharmacists’ practice in this respect. Face and content validation for the interview guide were conducted. The research team reviewed the interview guide to ensure its clarity, relevance, and reasonableness. A faculty member with research experience in medication use and misuse checked the content of the interview guide to ensure that it was logical and balanced. The interview guide was piloted with two community pharmacists. Piloting the interview guide indicated that conducting the interviews in the participants’ first language (Arabic) rather than in English would allow participants to express their thoughts easily and freely and provide more in-depth data. In addition, it showed the need to refine the interview guide with more probing questions. Subsequently, the interview guide was translated into the Arabic language.

### Sampling and recruitment

As the research team was unable to purposively recruit pharmacists from other local and national chains or independent pharmacies, convenience sampling was used to recruit pharmacists who expressed their willingness to participate in the study after receiving the initial invitation sent by the company’s regional supervisor. However, we considered the area of practice (urban vs. rural), working experience as community pharmacists, and qualifications to ensure diversity in the experiences of pharmacists. Out of the 38 pharmacists working in (X) chain pharmacies across the Al Baha region, 22 agreed to participate in the study. Sampling and recruitment of participants continued until data saturation was reached.

### Data collection

Pharmacists who agreed to participate in this study were emailed a participant information sheet (PIS) stating the study’s objective and expected time commitment. They were asked to inform the research team about the suitable date, time, and location for conducting the interview. The participants were sent an overview of the questions in advance to allow for reflection before conducting the interview. For safety purposes during the COVID-19 pandemic, the first six interviews were conducted face-to-face, and the remaining were conducted over the phone. For the face-to-face interviews, the interviewer (MA) met the pharmacists at their workplace. Interviews were conducted in Arabic (the National language of Saudi Arabia) and took, on average between 19 and 45 min. Interviews were conducted between March and July 2020 and were digitally audio-recorded. Using the approach proposed by Francis et al. (2010), data saturation was ensured after no new themes emerged from the last three consecutive interviews (Francis et al., 2010).

### Data generation and analysis

Data were transcribed verbatim and independently checked for transcription accuracy. Participants were permitted to review their transcripts to allow further review and editing of the transcripts. The interview transcripts were translated into English by a certified company. To ensure the translation accuracy, an independent expert in Arabic-English translation checked the translation of three randomly selected interviews. The translated interviews were then entered into the qualitative software NVivo 12 plus (QSR International) for initial coding. The analysis was interpretative, focused on the participants’ experience, and relied on the interaction between the researcher and the data. Thematic analysis was guided by the six steps developed by Braun and Clarke, including familiarisation with the data, coding, generating initial themes, reviewing themes, defining and naming themes, and writing up (Braun and Clarke, 2006). Data familiarisation was conducted simultaneously with data collection through listening to the recordings and reading the produced transcripts. Transcripts were read thoroughly to shape initial impressions and develop potential themes. The first author (MA) coded each transcript and created a set of codes pursuant to careful reading of all transcripts. A randomly selected sample of interviews were coded by another investigator of the research group (ZJ). Codes from individual transcripts were checked line-by-line, then compared, discussed, and changes were made as applicable until a consensus was reached. Categories were derived from combining codes to create an analytical framework, and the primary themes were formed. The analytical framework was refined iteratively over the analysis through discussions among the research members experienced in qualitative research (MH and SA).

To ensure rigour and trustworthiness in our study, strategies like member checking, reflexivity, and a detailed audit trail were used.

### Ethics

The ethics approval for this study was obtained from the University of Birmingham Science, Technology, Engineering, and Mathematics Ethics Committee (Ref# ERN_19_1636). Permission and approval were also obtained from the participating chain pharmacy. All participants signed a paper or electronic informed consent, agreed to audio record the interview, and gave verbal consent prior to conducting the interviews.

## Results

**FIGURE 1:**
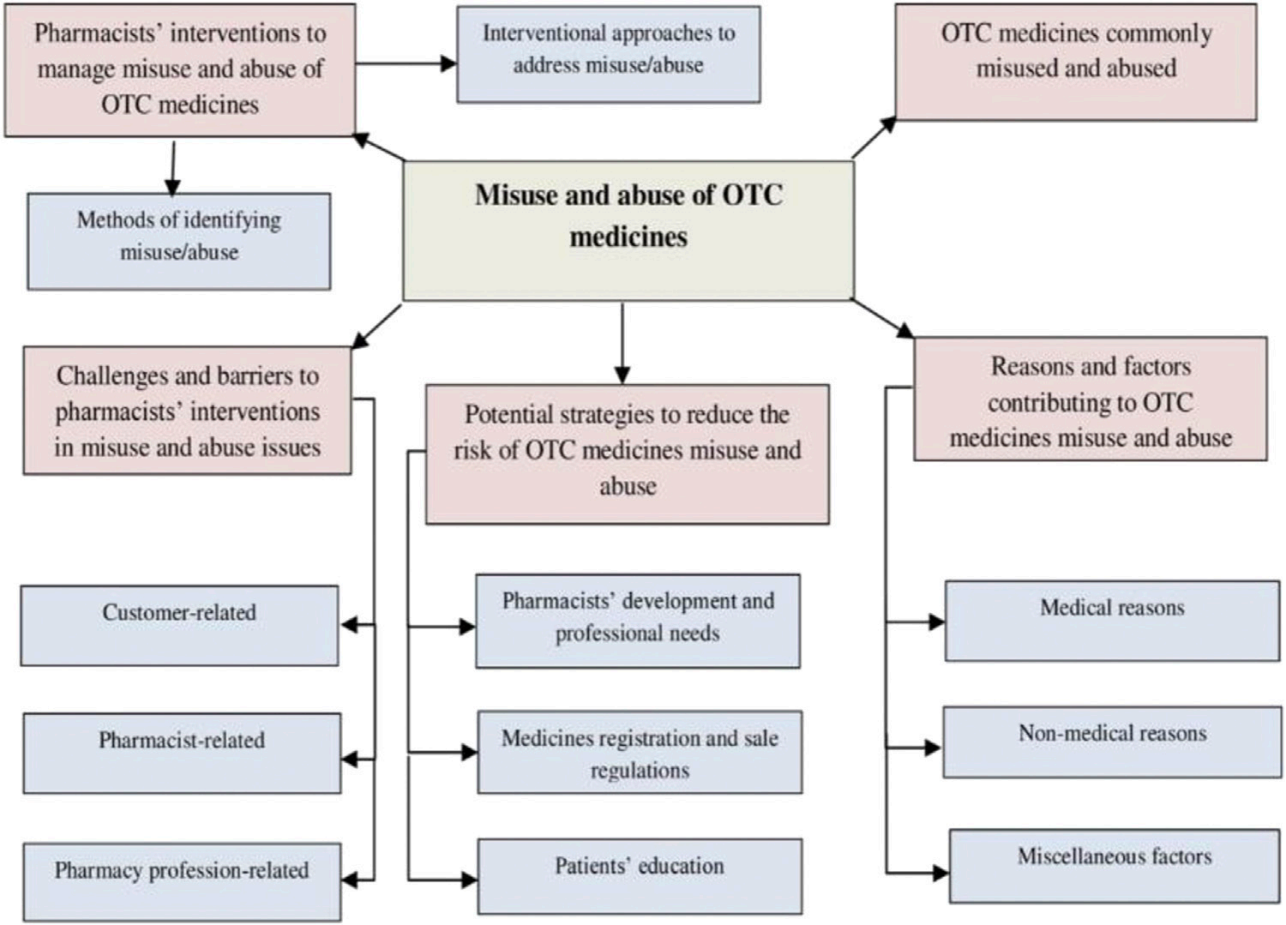
Emerged themes and subthemes.

A saturation of data was reached after interviewing sixteen pharmacists. All the respondents were male, while the majority had work experience ranging from 5 to 10 years and worked in pharmacies located in urban areas. Further demographic details of the participants are presented in Table 1. The coding process and subsequent analysis of the findings generated five main themes, including the commonly misused and abused OTC medicines, reasons and factors contributing to misuse and abuse of OTC medicines, pharmacists’ interventions to manage misuse and abuse, challenges and barriers to pharmacists’ interventions in misuse and abuse issues and potential strategies to reduce the risk of OTC medicines misuse and abuse and improve pharmacists’ practice (Figure 1). Pseudonyms were developed to show information relevant to each participant, such as pharmacist participant number (e.g., PH 1); pharmacy location (urban ‘ur’ or suburban ‘sub ur’ or rural ‘ru’); and the number of years the participant had been working as a pharmacist (e.g., 5 years).

**TABLE 1:** Demographic details of participants.

Characteristic	N (%)
Gender	
Male	16 (100)
Education	
B.Sc	15 (94)
PharmD	1 (6)
Years of practice	
Less than 5 years	4 (25)
5–10 years	8 (50)
11–15 years	3 (19)
More than 15 years	1 (7)
Pharmacy location	
Urban area	10 (63)
Suburban area	3 (19)
Rural area	3 (19)

### Theme 1: OTC medicines commonly misused and abused

The participating pharmacists nominated various OTC products commonly misused/abused or suspected to be misused/abused by their customers. These products largely belonged to the following classes; sedative antihistamines, dextromethorphan-based cough products, codeine-based analgesics, non-codeine-based analgesics, cortisone-based skin preparations, and nasal decongestants. All quotes are [sic].

*“Well. At first place comes (Diphenhydramine combined with Paracetamol), then comes (codeine-based analgesics). We may place Dextromethorphan in the second place, and then come (codeine-based analgesics) in the third place. We may place the analgesics in the fourth order because you cannot judge whether or not the customer is a misuser”* PH8 Sub Ur (11 yr).

Most pharmacists reported that they predominantly encounter misuse/abuse among male customers as they are frequent visitors to their local pharmacies requesting medicines for themselves or their female partners.

*“Our pharmacy, here on the South Line location and that’s why the majority of the customers I have, may be 80–90%, of them are males”* PH11 Ur (6 yr)

However, specific products such as cortisone-based topical preparations were reported to be heavily abused by women for skin whitening purposes.

*“For example, Cortisone cream is recently misused by some women in a skin whitening mixture because of the cortisone side effects is that it can bleach skin”* PH6 Ur (9 yr).

Regarding customers’ age, products with abuse potential were mainly perceived to be abused by young people, while misuse was more experienced in older people, particularly with analgesics

*“Painkillers are mostly misused by the old-aged while these antihistaminic drugs and these containing codeine like [codeine-based painkiller] are mostly abused by young adults”* PH6 Ur (9 yr).

Community pharmacists also noticed some patterns in the shopping behaviour of prospective abusers of OTC medicines. For example, customers craving sedative antihistamines frequently visits the pharmacy at night.

*“for (Diphenhydramine) and (codeine), people come at the end of the night while consumers of other things may come in the middle of the day and the afternoon at the crowded times"* PH16 Ur (1 yr).

Others try to visit the pharmacy when it is busy to avoid being questioned by pharmacy staff.

*“they come at the time when pharmacy is crowded with customers exploiting, for example, that I have no free time to spend with them. They aspire to win their request and leave right away"* PH10 Ru (9 yr).

### Theme 2: Reasons and factors contributing to OTC medicines misuse and abuse

Pharmacists perceived several medical and non-medical reasons and other factors precipitating OTC medicines misuse/abuse. Pharmacists perceived that managing ongoing medical conditions such as toothache, migraine, and osteoarthritis is the main reason for customers to consume higher doses of OTC analgesics or use them for a more extended period of time.

“they misuse very big quantities of them (painkillers) to relieve toothache to the extent that someone may take 1200 or 1800 mg of (Ibuprofen) per day. But you do not talk about for one-time use but for long-term use” PH5 Ru (7 yr).

Numerous non-medical reasons for abusing OTC medicines were also reported such as abusing codeine-based analgesics or dextromethorphan-containing products for recreational purposes and sedative antihistamines to induce sleep.

*“secondly, we are also asked, of course, about [codeine-based painkiller]. It is either used for getting high which has nothing to do with the prescribed medication or for its analgesic effect for a long time"* PH5 Ru (7 yr).

Two pharmacists reported a more dangerous practice with sedative antihistamines as some parents administer syrups to their children to induce sleep.

*“Others may misuse them to get sedated and that's why they resort to taking Chlorpheniramine maleate in the form of pills or syrups"* PH13 Ur (8 yr).

Female customers in particular abuse skin creams containing cortisone for skin whitening purposes and sometimes mix them with Hydroquinone and Tretinoin.

*“Despite the availability of alternatives, [Cortisone containing cream] is still famous among some people who misuse it in mixtures for the purpose of whitening skin as it happens when used with Hydroquinone and Retinoid in this famous mixture"* PH10 Ru (9 yr).

Among customer-related factors, seeking unprofessional advice from others and searching on the internet for information were the most commonly reported contributor to misuse/abuse

*“look, the reason could be that one of his relatives tells him about that medicine and how good it is, and how it helps him to sleep well, so he imitates him, or he uses it profusely so he will be misusing the medicine and he will get used to it like [product combines diphenhydramine and paracetamol] "* PH12 Ur (12 yr).

In addition, lack of awareness about medicines, low education, and cultural influence were other important factors reported by many participants. Pharmacists illustrated that highly-educated individuals appeared more amenable to accepting advice once problematic use of OTC medicines was identified.

*“Of course, educational level makes a great difference. When some customer is educated person, he listens to my advice and even thanks me. When the customer sometimes has low educational level, he can enter into an argument shouting impolitely at me in order to give him the medication and stop admonishing him"* PH9 Ur (8 yr).

Pharmacists reported that previous experience of using the medicine as well as previous medical prescribing urged customers to repeat using medicines when they experience the same symptoms without following up with the prescriber

*“Some patients may, for example, go to hospital and physician prescribes some medications including these OTC medicines for him. That patient by himself keeps repeating the dosage when having the same previous symptoms"* PH3 Ur (10 yr).

Pharmacists described the importance of peer pressure in driving individuals to behave similarly toward medicine use. For instance, pharmacy visitors from a specific nationality or the same age group, such as young individuals, share the same misuse/abuse practices.

*“many of these people of non-Arab nationalities especially Indians and Bengalis, sometimes ask about medicines for coughing, which is a cough suppressant like [dextromethorphan product] and [dextromethorphan product] which are very well-known"* PH14 Sub Ur (8 yr).

Pharmacists perceived that the inability to access General Practitioners (GPs) and hospitals due to cost entails some people using OTC medicines without proper medical diagnosis or supervision.

*“yes, the point of preferring to visit pharmacy to visiting a doctor has more than one reason, including the financial side that he is not able to go to hospital and pay for the doctor's costs"* PH6 Ur (9 yr).

Pharmacists reported two factors related to OTC products that precipitate their misuse/abuse. Firstly, advertising products on television and social media, and secondly, affordable price of generic medicines with abuse potential.

*“let me give you an example.[Xylometazoline] has some specific usages; it must be used for 5 days only at maximum; otherwise, it can cause a rebound effect which has many more harmful effects than the benefits. It is actually advertised on TV and as a result, people ask for it a lot"* PH7 Sub Ur (17 yr).

### Theme 3: Pharmacists’ interventions to manage misuse and abuse of OTC medicines

Pharmacists described how they identify misuse/abuse incidents through customers’ behaviours and attitudes and procedures they have in place. Most pharmacists reported that misusers/abusers could initially be judged by observing their facial expressions, physical appearance, and reactions during conversations

*“another sign is the behavior some of those people show when first visiting pharmacy; they may a little bit confused or perplexed in his speech or movements. You may find them apparently unnatural”* PH9 Ur (8 yr).

Many pharmacists also regarded frequent visits requesting the same product, requesting a large quantity at once, visiting the pharmacy during busy times, exploiting the crowd to avoid being asked by pharmacists, and refusing any alternative as usual behaviours aiding in the identification of misuse and abuse incidents.

*“the pharmacy here, especially in the Al-Baha region, is considered a neighborhood pharmacy, so customers almost daily frequent at it. Therefore, I can mostly identify them by frequently visiting pharmacy and asking for the same drug”* PH5 Ru (7 yr).

Few pharmacists indicated that some customers disclose to them the non-legitimate use of the medication.

*“when they visit me, they tell frankly that they want that comforting syrup, such as [product combines pseudoephedrineand and triprolidine], for example, or any antihistaminic syrup”* PH6 Ur (9 yr).

Concerning the procedures used by pharmacists to identify problematic use with OTC medicines, nearly half of pharmacists specified the pharmacy mnemonics WWHAM (which stands for five questions W = Who is the patient? = What are the symptoms? H = How long have the symptoms been present? A = Action taken, M = Medication being taken) as the main procedure for gathering essential clinical information from customers.

*“you should use the regular WWHAAM questions with anyone visiting you. Before dispensing the medications for him, you should ask why he gets the medications and what the symptoms are”* PH13 Ur (8 yr).

Some pharmacists stated that they get notified about misuse/abuse incidents by their colleagues or a nearby pharmacy

*“In case some of them frequently request the medication from other pharmacies and we are told and warned of them that they were, for example, searching Al-Baha city for getting the same medication”* PH16 Ur (1 yr).

Regarding the actions taken by pharmacists to address misuse/abuse, advising customers on the appropriate use of medications and the wise handling of objections were the initial actions taken by most pharmacists.

*“Someone may have recommended for the visiting customer a medication for cough that the customer even does not know anything about it. The customer may not be an addict at all; in that case, you can counsel him about it” PH14 Sub Ur (8 yr)*.

However, offering safe alternative products was the most reported action by pharmacists when the requested medications were being surely misused/abused

*“I have the Codiene shown outside because it is among the OTC medications. As pharmacist, I try to play my role in order to know why the customer is taking it and try to shift him to another alternative.”* PH5 Ru (7 yr).

Pharmacists dealt with those who used OTC medicines for medical reasons but in wrong ways by providing a limited quantity of the product or reducing its dose and frequency

*“I can decide the action taken; I can urge him to decrease the used dosage so as to minimize the side effects and thus reduce the rate with which he takes the drug in the wrong way”* PH5 Ru (7 yr).

Community pharmacists, when left with no other option to prevent misuse/abuse of OTC medicines, had to refuse to sell the product or deny its availability. Community pharmacists would also notify their colleagues or the nearby pharmacies about customers with the potential of abusing OTC medicines, especially pharmacists working in rural areas who had better communication with local pharmacies of different groups, whereas those working in urban areas exchange information only with the same chain pharmacies.

*“I am here talking about Al-Aqiq as we created a group on WhatsApp for our group of pharmacies. That group not only includes (Our company), but also includes (Another chain pharmacy) as they are four pharmacies or four companies. We have a group for all of them so that we can tell each other what happens”* PH4 Ur (10 yr).

As a precautionary procedure, pharmacists kept products with misuse/abuse potential out of customers’ sight to prevent abusers from picking them directly from the shelves

*“generally, I keep these well-known abused/misused drugs, such as [product contains diphenhydramine and paracetamol], [codeine-based painkiller] or [topical cortisone] behind the counter so that no one else can see them”* PH10 Ru (9 yr).

Lastly, pharmacists stated that they often signpost customers requiring further care or those non-responsive to their advice to GPs and hospitals.

*“in case he was not to accept my advice, I would recommend visiting any physician so that he could have some examinations and blood tests”* PH8 Sub Ur (11 yr).

### Theme 4: Challenges and barriers to pharmacists’ interventions in misuse and abuse issues

Pharmacists reported multiple barriers and challenges relating to customers, pharmacists, and the pharmacy profession that hinder their interventions. Failure to change customers’ perceptions about OTC medicines was the most common challenge mentioned by pharmacists

*“we offer the alternative, but almost 98% of people do not get convinced” PH11 Ur (6 yr).*

*“On the other hand, there are some experienced customers who can answer all questions in such a way that convince us to dispense the medications for him. I mean he can circumvent to take the medications and that happens a lot”* PH6 Ur (9 yr).

Pharmacists were concerned about raising the issue of misuse/abuse among women and the elderly especially.

*“the first main challenge is keeping the privacy of females; I cannot investigate deeply the whole matter. I would talk to her as a pharmacist; in case there was rigidity from the person before me, I would not enter into this debate or in the discussion”* PH5 Ru (7 yr).

Some pharmacists complained that the customer would easily obtain the medicine from another pharmacy once their request was denied.

*“In fact, we did not; he went, unfortunately, to the other pharmacies and they were selling quantities of the medications he wanted”* PH4 Ur (10 yr).

One pharmacist mentioned that purchasing medicines online limits the contact with the customer and gives no opportunity to identify any problematic use*.*

*“up till now, we and my colleagues can control the question of the OTC abuse/misuse because we want to do that. But we have some electronic service in [our company] which can enable the visitor to shop online. It also enables him to know his credit in the pharmacy and it is his right to order the medications. In this case, you are forced to get the medications ready for shipment for him”* PH14 Sub Ur (8 yr).

Pharmacists stressed that identifying misuse/abuse in new customers was challenging. This was especially challenging among pharmacists with limited experience and little sociocultural awareness.

*“As for us working here in a community pharmacy and thanks for long experience, we have become very acquainted with most of our guests. Therefore, it is normal to have some unfamiliar customer whom you did not see before”* PH1 Ur (4 yr).

Concerning the work environment and pharmacist-related barriers, personal safety was the most often reported barrier. In particular, it limits those working in rural areas from dealing with aggressive customers.

*“of course, the first barrier is taking care of your own safety as a pharmacist or of the person you are dealing with. I mean, if someone in a state of drunkenness or intoxication with some illegal drug visited you to ask for some medication, then it would be natural for you to be afraid of clashing with him”* PH10 Ru (9 yr).

Workload was noted by many pharmacists as it forces them to reduce their contact time with customers during busy times

*“Moreover, workload and rush hour make me so occupied that I have no enough time to talk freely with the patient so as to completely advise him as possible as I can”* PH5 Ru (7 yr).

Pharmacists indicated that the lack of standardised protocol for OTC medicines sale and scarcity of training designed for abuse management limited their capability in managing abuse issues.

*“of course, there are no clear statements from the Ministry of Health concerning OTC medications. Thus, you cannot abstain from dispensing these medications for customers because the Ministry of Health cannot support you in case you do”* PH13 Ur (8 yr).

Only a few pharmacists considered business targets, lack of experience, and initiative to deal with abuse issues as barriers. A majority of pharmacists reported that drug distributors often pressured them to display products with misuse/abuse potential in a visible location for customers.

*“the salesperson or the one who are in the pharmacy waits for such pretext for selling more and more of analgesics”* PH2 Ur (4 yr).

Some pharmacists referred to the poor connection with other local pharmacies as limiting information sharing about medicines abusers

*“No, frankly speaking. There is no contact with other pharmacists who work in other pharmacies outside (X) group*” PH9 Ur (8 yr).

### Theme 5: Potential strategies to reduce the risk of OTC medicines misuse and abuse

Pharmacists perceived several strategies and professional development requirements to help mitigate OTC medicines misuse/abuse. Switching abuse susceptible medicines from OTC to prescription-only or behind-the-counter status by health authorities was considered the most robust strategy. For instance, rescheduling codeine-based analgesics to prescription-only status was suggested by most pharmacists.

*“Yes, codeine and Chlorpheniramine contained in [product X] are really supposed to be prescriptions only”* PH13 Ur (8 yr).

Half of the pharmacists perceived that developing a nationwide standard protocol for OTC medicines sales would uniform the procedures followed by all pharmacists. Similarly, pharmacists showed the need for training programs on abuse and dependence management and providing continuous informational updates about abuse issues

*“You may encounter dangerous situations in which you can deal with some serious addicted person. You may not know how to deal with him. That’s why it is so important to learn- even if you have reached a very high degree of experience- how to manage such people and how to guide and counsel them”* PH1 Ur (4 yr).

Pharmacists emphasised that raising public awareness about OTC medicines’ safe use is crucial to reducing their risks. Two pharmacists stressed that lessons could be learned from a previous campaign, which successfully raised public awareness about antibiotics’ rational use

*“that’s why when the media talked about antibiotics and their bad side effects, it affected people. That' s why I have told you earlier that there has to be an awareness among people, and it should not be by one side”* PH12 Ur (12 yr).

Monitoring the advertisement of OTC products *via* different media means was suggested by some pharmacists

*“of course, I see that it is necessary to restrictions to be imposed. I do not know whether or not they can be applied. When you make advertisements for some drug, you mean to say that it has no side effects. In Europe, they tell people about all pros and cons of the product. That is no applied here in Saudi Arabia”* PH4 Ur (10 yr).

Some pharmacists suggested regulations to determine a maximum pack size for medications susceptible to misuse/abuse and restrict the number of packs at the time of sale

*“of course, it is possible to reduce number of pills in each medication box. You as customer should get but one box. That can already help in minimizing abuse”* PH7 Sub Ur (17 yr).

Other strategies less often mentioned by pharmacists were enhancing cooperation between local pharmacies, allowing pharmacists to access patient medical records, tracking the sale of medicines to identify the frequently misused/abused medicines, and convening workshops for pharmacists and GPs to share experiences about medicines abuse and inform GPs prescribing decisions.

## Discussion

The aim of this qualitative study was to explore the views and experiences of the Saudi community pharmacists towards OTC medicines misuse and abuse, specifically aiming to identify the implicated medicines, reasons, and contributing factors, the role of pharmacists and potential risk-mitigating strategies. In the present study, the common OTC medicines reported by pharmacists to be misused/abused were first-generation antihistamines, codeine-based analgesics, and antitussive preparations. These findings are in line with previously published literature in other countries (Wazaify et al., 2006; Cooper, 2013a; Abood and Wazaify, 2016; Wazaify et al., 2017). Pharmacists stressed that OTC analgesics, including codeine-based, nonsteroidal anti-inflammatory drugs (NSAIDs) and paracetamol, are misused to manage pain, especially by the elderly. Therefore, the potential risks in such vulnerable groups are highly anticipated due to comorbidities and polypharmacy. Other studies have also identified painkillers as the most common OTC medicines misused by older people and their chronic use was the most common form of misuse (Elhoseeny et al., 2013; Stone et al., 2017). On the other hand, abusing OTC medicines to obtain mind-altering effects was usually encountered in young people. Sometimes, such medicines are used concurrently with illegal substances or as alternatives to the inaccessible abused prescription medications.

In the present study, pharmacists often attribute misusing OTC medicines by customers to not seeking professional advice. Instead, people tend to take advice from friends and family or use the internet as a source of information about medicines. In an Egyptian study, pharmacists illustrated that customers do not seek advice on OTC medicines as the majority trust the safety of these medicines, and the others believe that seeking advice is unnecessary (Elhoseeny et al., 2013). Seeking advice for self-medication from friends and parents is the secondary source of information in Middle Eastern countries (Khalifeh et al., 2017). Pharmacists perceived that misuse/abuse is influenced by geographic, educational, cultural, and ethnic disparities among customers. An earlier cross-sectional survey conducted in the United States revealed that people with low income and low education show significantly less risk awareness about OTC and prescribed NSAIDs, with significant racial/ethnic disparities in risk awareness, communication, and behaviour (Fry et al., 2007). Among OTC product-related factors, direct-to-consumer advertising (DTCA) was perceived by pharmacists as strongly influencing the abuse of certain products and making consumers unresponsive to professional advice. Australian pharmacists believed that DTCA of OTC medicines disempowered them, undermining their role in protecting the community from inappropriate medicine use (Chaar and Kwong, 2010). Pharmacists also considered the affordable price of OTC medicines with abuse potential to drive abuse among young people. In contrast, the high cost of healthcare services necessitates low-income patients to use OTC products for long periods without a proper medical diagnosis. The low price and availability of OTC cough mixtures facilitated the recreational use among young people in China and the abuse of topical corticosteroids among women in India (Lam et al., 1996; Rathi, 2006). Pharmacists deemed the past experience of using the medicine and previous medical prescribing to precipitate frequent use for recurrent symptoms without seeking professional consultation. Previous or ongoing medical prescribing was associated with abusing OTC codeine by some users (Cooper, 2013b).

The common ways reported by pharmacists to identify misuse/abuse are, to some extent, similar to what has been reported in other studies (Eickhoff et al., 2012; Cooper, 2013a; Barrett and Costa, 2018). Pharmacists initially judge abusers by observing their behaviours and attitudes. In the United Kingdom, pharmacists commented that behaviours such as avoiding eye contact with the pharmacist, being agitated, and giving inconsistent answers are indications helping identify those abusing codeine analgesics (Carney et al., 2016). Pharmacists in the present study emphasised the use of the specific pharmacy mnemonic WWHAM to gather clinical when high-risk OTC medicines are requested. However, such standardised approaches do not necessarily improve consultation performance, possibly because the collected information is not always adequate for the decision-making process (Sinopoulou et al., 2019).

Pharmacists respond differently to each misuse/abuse situation. Resolving the situation through counselling, providing a limited quantity of the product, reducing the dose and frequency, and offering alternatives are actions usually taken with customers who misuse medications. Those customers generally accept the pharmacist’s advice. However, pharmacists require to take further actions against those abusing medicines for non-medical purposes, including refusing the sale or denying the availability of the product, referral to physicians, and notifying fellow pharmacists. It was shown that pharmacists successfully addressed 73.3% of OTC medicines-related problems, including misuse/abuse, in the pharmacies with no need for referral, while counselling and drug switching were the most commonly made interventions. (Ylä-Rautio et al., 2020).

Although pharmacists showed readiness to identify and resolve misuse and abuse issues, they face multiple barriers to intervening in such issues. Pharmacists face customers’ objections when medicine shifting is the appropriate choice to tackle the problematic use. Customers tending to abuse medicines for illicit purposes showed more resistance to medicine shifting. Also, those customers usually use different scenarios to circumvent obtaining their medicines. In a United Kingdom study, pharmacists showed that younger customers abusing OTC laxatives respond more defensively when questioned about the intended purchase as they think that they have the right to pick a General Sale List (GSL) medicine without being questioned by a pharmacist (Kelly and White, 2016). Pharmacists could refuse or deny the availability of the product when abuse is confirmed. However, customers can obtain the medicine easily from another pharmacy. Pharmacists can fairly identify and resolve misuse/abuse issues with their regular customers, but it is not the case with irregular ones. This advocates that loyalty and a good customer-pharmacist relationship could result in better outcomes when pharmacists intervene to tackle problematic use. Among barriers relevant to pharmacists and the work environment, lack of initiative to monitor the inappropriate use of OTC medicines and prioritising business interests could result in higher abuse opportunities. Nevertheless, workload, high-stress prescription processing, and low staffing make pharmacists less pharmacovigilant when the intervention is significant in preventing OTC medicines’ misuse/abuse (Sansgiry et al., 2017).

Worldwide, several strategies and programmes have been considered to mitigate the misuse and abuse of prescription medicines and in turn little attention paid to OTC medicines. In the present study, pharmacists highlighted the importance of standerdising national protocol to facilitate their role in OTC medicines counselling and provision. At present, pharmacists use their own informative guidelines to support the advice-giving role. Such protocols are intended to guide pharmacists in identifying ailments, selecting medicine, counselling, following up with the patient, and referring for further care (Cavaco and Pereira, 2012). However, extending such protocol to include medicines abuse management could be beneficial. Pharmacists raise the need for specific training programmes in the area of abuse management as well as access to informational updates about abuse issues. An expert panel rated providing training for pharmacists and pharmacy staff as the most important and effective approach to reducing the inappropriate use of OTC medicines (McBride et al., 2003). In terms of regulations, pharmacists call for rescheduling OTC medicines with abuse potential into prescription-only or into a third-class that requires pharmacists’ assessment upon dispensing. Currently, in Saudi Arabia, medicines are classified into prescription-only and OTC medicines. However, a third class of medicines, those with abuse potential, are currently defined in several countries, such as the pharmacist-only list in the United Kingdom and the behind-the-counter class of medicines in the United States. Medicines in this class are provided upon the pharmacist’s assessment after considering the required tests, preliminary screening, and appropriate counselling (Sansgiry et al., 2017). Moving codeine-combined analgesics from OTC to the pharmacists only category in Ireland and to prescription-only in Australia resulted in a misuse rate reduction (Cairns et al., 2016; Kennedy et al., 2019; Cairns et al., 2020). Because of its notable contribution to abuse, pharmacists emphasised that authorities should monitor the direct advertising of OTC products. Banning public advertising in addition to restricting pack sizes, limiting product visibility and customer self-selection, and increasing visibility of warnings on labels have been utilised in other countries to reduce codeine abuse (VAN and NORMAN, 2016). Eventually, pharmacists proposed raising public awareness about the safe use of OTC medicines. As some of the reported misuse/abuse practices in this study were attributed to illiteracy and lack of knowledge about medicines, considering health education is imperative to improve public knowledge about medicines.

### Study limitations

There are some limitations to this research. First, the method of interviewing participants varied, with six pharmacists interviewed face-to-face and nine by telephone. This is because the interviewer was not able to meet face-to-face with the remaining participants due to COVID-19 restrictions. Consequently, this could have impacted the quality of the data collected from interviewees over the phone since the interviewer could not grasp the participants’ body language. On the other hand, the increased sense of anonymity may have improved the quality of the data collected. Second, although the recruitment of participants considered a range of pharmacists working in urban, suburban, and rural areas, the study was conducted within one chain pharmacy in one region. Therefore, findings may not reflect the experiences of pharmacists working in independent or other chain pharmacies across the country. Third, medicines that are licenced in Saudi Arabia as OTC and the regulations for their sale may differ from those available in other countries. As a result, the study’s findings cannot be generalised to other countries with different healthcare systems and drug regulations.

## Conclusion

This study has provided unique insights into community pharmacists’ experiences and views regarding the misuse and abuse of OTC medicines. Pharmacists identified the commonly misused and abused OTC medicines amongst different sets of pharmacy customers besides the reasons and factors precipitating the problematic use. Pharmacists also described the current strategies adopted to identify and manage misuse/abuse cases. However, they raised multiple challenges and barriers to effectively managing the misuse/abuse of OTC medicines. Pharmacists proposed several strategies with regard to OTC medicine regulations, pharmacists’ professional needs, and patient education to mitigate the risk of OTC medicines misuse/abuse.

## Data Availability

The raw data supporting the conclusions of this article will be made available by the authors, without undue reservation.
